# Bacterial Manipulation of the Integrated Stress Response: A New Perspective on Infection

**DOI:** 10.3389/fmicb.2021.645161

**Published:** 2021-04-22

**Authors:** Alex Knowles, Susan Campbell, Neil Cross, Prachi Stafford

**Affiliations:** Biomolecular Sciences Research Centre, Department of Biosciences and Chemistry, Faculty of Health and Wellbeing, Sheffield Hallam University, Sheffield, United Kingdom

**Keywords:** bacteria, ISR, eIF2alpha, infection, immunity, stress

## Abstract

Host immune activation forms a vital line of defence against bacterial pathogenicity. However, just as hosts have evolved immune responses, bacteria have developed means to escape, hijack and subvert these responses to promote survival. In recent years, a highly conserved group of signalling cascades within the host, collectively termed the integrated stress response (ISR), have become increasingly implicated in immune activation during bacterial infection. Activation of the ISR leads to a complex web of cellular reprogramming, which ultimately results in the paradoxical outcomes of either cellular homeostasis or cell death. Therefore, any pathogen with means to manipulate this pathway could induce a range of cellular outcomes and benefit from favourable conditions for long-term survival and replication. This review aims to outline what is currently known about bacterial manipulation of the ISR and present key hypotheses highlighting areas for future research.

## Introduction

The relationship between microbes and hosts has shaped almost every aspect of microbial and mammalian evolution. This association is formed through an extensive series of interactions, some of which are beneficial, whilst others pose pathogenic threat. The immune system, consisting of innate and adaptive or acquired immunity, is a highly complex network enabling the human body to detect and determine the fate of foreign entities (Nicholson, 2016). During the first line of defence against pathogens, the host cell utilises the innate immune system to activate extensive signalling cascades in a concerted effort to defend against pathogenicity. These include activation of host pattern recognition receptors such as toll-like receptors (TLRs; Wright et al., 1989; Hoshino et al., 1999; Kumar et al., 2009) and Nod-like receptors (Opitz et al., 2005; Hasegawa et al., 2006), which detect structural bacterial features, termed pathogen-associated molecular patterns (Lai and Gallo, 2008; Davis et al., 2011). The resulting induction of pro-inflammatory cytokines enables host cells to initiate both intracellular and extracellular mechanisms to protect the cell and surrounding tissues (Lai and Gallo, 2008; Davis et al., 2011). These result in inflammation and the subsequent activation of macrophage- and neutrophil-mediated bacterial clearance at the infection site (Zhou et al., 2019). In some cases, autophagic responses are also triggered to remove foreign bacteria, such as *Salmonella* (Wild et al., 2011), with the host cells internalising the bacterium into double-membraned vesicles, termed autophagosomes, which are subsequently targetted for lysosomal degradation, thereby removing the foreign bacterium (Bah and Vergne, 2017).

To evade host-mediated innate immune responses, bacterial pathogens are also constantly evolving and developing mechanisms to ensure persistence within host cells and gain evolutionary success. Such mechanisms include antigenic variation (Saunders, 1990), inhibition of the humoral immune response by recruitment of complement inhibitors (Meri et al., 2013), direct interaction with complement components (Amdahl et al., 2013), evasion of autophagic responses (Ogawa et al., 2005), and residing in immune-privileged sites (Young et al., 2002). These strategies ultimately allow the bacteria to avoid detection and induce conditions favourable for bacterial survival and successful proliferation (Young et al., 2002).

In recent years, a group of highly conserved cellular pathways, collectively termed the integrated stress response (ISR), has gained increased interest in relation to host–pathogen interactions (Pakos-Zebrucka et al., 2016). The ISR, which can respond to a variety of stimuli, has been implicated in controlling the tight balance between cellular survival and death during adverse conditions, with a body of evidence implicating cross-talk between the ISR and viruses, forming a key mechanism of viral pathogenesis (Rabouw et al., 2020). The aim of this review is to explore to what extent bacteria have exploited these stress response pathways to overcome cell defences. Given that the ISR functions as a master regulator of cellular fate, understanding to what end bacteria can manipulate these pathways will allow for a better understanding of their disease pathology. Furthermore, as antibiotic resistance is on the increase, a better understanding of these host–microbe interactions may help identify novel candidate therapeutic targets.

## The Integrated Stress Response

**FIGURE 1:**
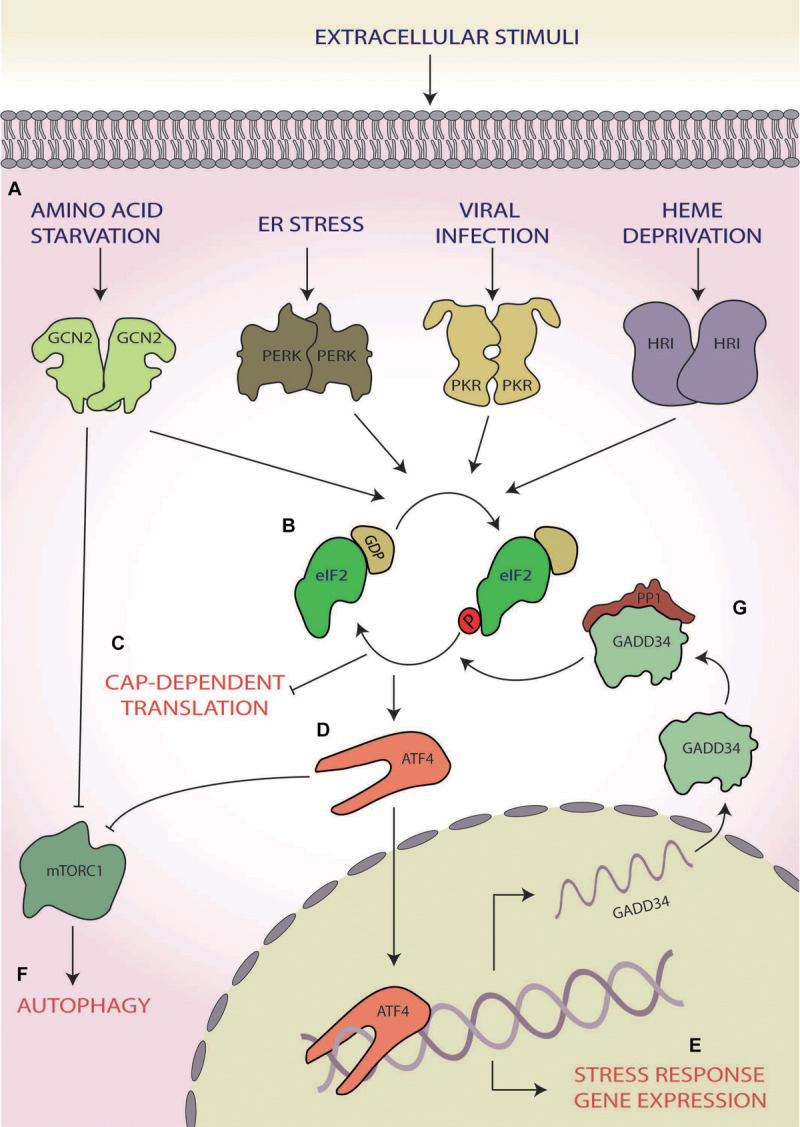
The integrated stress response (ISR). **(A)** A range of cellular stress stimuli activate one of four stress response kinases, general control non-depressible 2 (GCN2), protein kinase R-like endoplasmic reticulum (ER) kinase (PERK), protein kinase R (PKR), and heme-regulated inhibitor HRI kinases, which **(B)** phosphorylate eukaryotic initiation factor 2 alpha (eIF2α). **(C)** This results in abrogation of canonical translation initiation, **(D)** which selectively upregulates the translation of ISR effector mRNAs, such as activating transcription factor 4 (ATF4). **(E)** These effectors bind to and target genes involved in cellular reprogramming for expression. **(F)** GCN2 and ATF4 also both induce autophagy via inhibition of mammalian target of rapamycin complex 1 (mTORC1). **(G)** If stress is overcome, the stress-inducible phosphatase growth arrest and DNA damage-inducible protein (GADD34) dephosphorylates eIF2α, returning homeostatic translation initiation and terminating the ISR.

Within eukaryotic cells, the ISR is a mechanism that, in response to changes in either intracellular or extracellular conditions, has the capability of switching between cellular survival or inducing cell death by triggering a range of signalling cascades (reviewed by Pakos-Zebrucka et al., 2016). Stimuli can include both physiological and pathological changes and once triggered results in the reduction of global protein synthesis, allowing the cell to focus energy into overcoming stress (Brostrom and Brostrom, 1997) mediated via the phosphorylation of eukaryotic translation initiation factor 2 alpha (eIF2α; Siekierka et al., 1982; Donnelly et al., 2013; Figures 1A–C). However, during ISR activation, there is also increased translation of a selection of stress response mRNAs via non-canonical translation (Ryoo and Vasudevan, 2017). This includes mRNAs coding for transcription factors, such as activating transcription factor 4 (ATF4), C/EBP homologous protein (CHOP), and growth arrest and DNA damage-inducible protein (GADD34), which act as effectors of the ISR (Lee et al., 2009; Palam et al., 2011; Hinnebusch and Lorsch, 2012) specifically upregulating the expression of genes involved in cellular reprogramming under stress conditions (Karpinski et al., 1992; Harding et al., 2003; B’chir et al., 2013; Figures 1D,E).

Of the ISR effectors, ATF4, a basic leucine zipper transcription factor, is the best studied (Karpinski et al., 1992; Vallejo et al., 1993; Ameri and Harris, 2008). Once activated, ATF4 regulates the expression of genes involved in stress responses, amino acid (AA) synthesis, metastasis, angiogenesis and differentiation, allowing for a stress-specific cellular response (Ameri and Harris, 2008). During hypoxia, endoplasmic reticulum (ER) stress, and AA starvation, ATF4 also upregulates transcripts involved in autophagy (Rzymski et al., 2010; B’chir et al., 2013; Deegan et al., 2015). One mechanism by which this is achieved is the inhibition of mammalian target of rapamycin (mTOR) complex 1 (mTORC1) via translational upregulation of regulated in development and DNA-damage response 1, which functions to activate autophagic responses (Whitney et al., 2009; Kroemer et al., 2010; Dennis et al., 2013; Figures 1D,F).

Through the action of ATF4, the ISR can induce cell death via upregulation of downstream targets including the transcription factors CHOP and ATF3 (Puthalakath et al., 2007). One mechanism for this function is via CHOP increasing the expression of additional pro-apoptotic factors from the Bcl-2 homology 3-only group of the Bcl-2 family (Puthalakath et al., 2007; Galehdar et al., 2010). It has also been suggested that ATF4 and CHOP may interact directly to form heterodimers to heighten the expression of pro-apoptotic genes, such as Bim (Teske et al., 2013).

However, the ISR can also induce cellular survival and overcome the stress. In this case, upon cessation of stress, GADD34 activates protein phosphatase 1 (PP1), which dephosphorylates eIF2α (Connor et al., 2001; Novoa et al., 2001), thus terminating the ISR and returning the cell to homeostatic translation (Figure 1G; Novoa et al., 2001, 2003). As such, the ISR can induce the directly opposing outcomes of cellular survival or death.

Whilst the ISR can be initiated by multiple stimuli (e.g. AA starvation, ER stress, viral infection, and heme deprivation), the point of convergence of this response hinges upon the abrogation of canonical translation initiation via the phosphorylation of eIF2α at serine 51 (Siekierka et al., 1982; Donnelly et al., 2013; Figure 1). During homeostatic translation initiation, eIF2 in its active-GTP bound form associates with the initiator methionyl tRNA (tRNA_*i*_^*Met*^) to form a ternary complex. Upon AUG recognition, the tRNA_*i*_^*Met*^ is released, and eIF2-GTP is hydrolysed to eIF2-GDP (Hinnebusch and Lorsch, 2012). To enable subsequent rounds of translation, eIF2-GTP is regenerated by the guanine nucleotide exchange factor (GEF) eIF2B (Price and Proud, 1994; Jennings et al., 2013). The activation of the ISR and the subsequent phosphorylation of eIF2α at serine 51 block eIF2B GEF activity and result in a deficit of cellular eIF2-GTP and subsequently the ternary complex (Kenner et al., 2019), leading to the shutdown of most mRNA transcripts (Figures 1B,C). Some of these stalled complexes of translational machinery and transcripts consisting of both proteins and RNAs are sequestered into dynamic, phase dense, cytoplasmic foci termed stress granules (Nover et al., 1989; Anderson and Kedersha, 2002). Stress granules can form within minutes and dissolve at a similar pace upon stress cessation (Kedersha et al., 2000). Due to their dynamic nature, they require ongoing retrograde transport of stalled translational machinery along functioning microtubules (Loschi et al., 2009). Functionally, stress granules play a key role in allowing molecules to be sorted for storage, degradation or for re-initiation of translation, thereby allowing for rapid sorting of transcripts once homeostasis is returned (Nover et al., 1989; Anderson and Kedersha, 2002).

To enact the core function of the ISR, eIF2α phosphorylation is mediated by a family of four serine/threonine stress response kinases (Wek et al., 2006). Whilst all four kinases share significant sequence similarity in their kinase domain (Donnelly et al., 2013), each contains a unique regulatory domain, allowing for differential regulation via distinct stressors (Meurs et al., 1990; Chen et al., 1991; Berlanga et al., 1998; Shi et al., 1998; Dong et al., 2000; Harding et al., 2000; Rafie-Kolpin et al., 2000). Protein kinase double-stranded RNA-dependent (PKR) also known as EIF2AK2 classically responds to double-stranded RNA generated during viral infections (Clemens and Elia, 1997). PRK has also been found to respond to oxidative and ER stress as well as cytokine signalling and reactive oxygen species (ROS; Cheshire et al., 1999; Ito et al., 1999; Ruvolo et al., 2001; Onuki et al., 2004; Nakamura et al., 2010; Anda et al., 2017). The protein kinase R-like ER kinase (PERK, EIF2AK3) forms one arm of a larger three-armed response to misfolded proteins in the ER, collectively termed the unfolded protein response (UPR; Walter and Ron, 2011). It is typically activated by ER stress, brought on by the accumulation of misfolded proteins in the ER lumen (Harding et al., 2000; Walter and Ron, 2011) and by changes to ATP and Ca^2+^ in the ER independently of misfolded proteins (Sanderson et al., 2010). PERK can also be activated by oxidative stress and hypoxia (Koumenis et al., 2002; Harding et al., 2003). General control non-depressible 2 (GCN2, EIF2AK4), the most highly conserved eIF2α kinase (Yang et al., 2000; Donnelly et al., 2013), is activated primarily by AA starvation (Wek et al., 1995) but can also been activated by ROS, viral infection and UV radiation (Berlanga et al., 2006; Grallert and Boye, 2007; Pyo et al., 2008). Heme-regulated inhibitor (HRI; EIF2AK1), a kinase mainly associated with protection against toxic globin aggregates in erythroid cells, is involved in protection against ROS induced by sodium arsenite as well as proteasome inhibition (Han et al., 2001; Lu et al., 2001; McEwen et al., 2005; Chen, 2007; Yerlikaya and DoKudur, 2008). Interestingly, to date, bacterial pathogens have been shown to activate all kinases with the exception of the viral specific kinase, PKR (Tattoli et al., 2012; Tsutsuki et al., 2016; Abdel-Nour et al., 2019).

## Bacterial Manipulation of the Integrated Stress Response

In recent years, it has become apparent that the ISR forms an integral part of the host innate immune response to pathogens (reviewed by Rodrigues et al., 2018). This is supported by studies showing that pathogens can induce eIF2α phosphorylation via PERK, GCN2 and HRI (Tattoli et al., 2012; Tsutsuki et al., 2016; Abdel-Nour et al., 2019). Given that the ISR plays a crucial role in controlling cellular fate during stress (Costa-Mattioli and Walter, 2020), pathogens with means to dampen or hijack the ISR pathway are likely able to influence cellular signalling and ultimately benefit from long-term survival and promote persistence of infection. Indeed, it is well-documented that viruses manipulate specific elements of the ISR during infection. Hepatitis C virus, Japanese encephalitis virus and human cytomegalovirus directly inhibit the viral specific eIF2α kinase PKR (Toroney et al., 2010; Tu et al., 2012; Ziehr et al., 2016), and Kaposi’s sarcoma-associated virus indirectly inhibits PKR via inhibition of its activator PACT (Sharma et al., 2017). Another point of ISR modulation displayed by viruses is to dampen eIF2α phosphorylation. Junin virus directly inhibits eIF2α (Linero et al., 2011), and hepatitis C virus activates GADD34 to dephosphorylate eIF2α during stress (Ruggieri et al., 2012).

However, recent evidence suggests that some bacterial species might also be manipulating the host ISR, inducing a variety of cellular outcomes. This review will focus on how five bacterial model organisms, Shiga toxin-producing *Escherichia coli*, *Shigella flexneri*, *Salmonella enterica* serovar Typhimurium, *Pseudomonas aeruginosa*, and *Porphyromonas gingivalis* can manipulate specific components of the ISR to gain control over cellular fate and immune signalling, creating an environment favouring bacterial viability, replication, and infection.

### Shiga Toxin-Producing *Escherichia coli*

Shiga toxin-producing *E. coli* O157:H7 (STEC) is a widespread pathogen presenting severe risk to human health, causing haemorrhagic colitis and haemolytic uremic syndrome (Riley et al., 1983; Ko et al., 2016). Annually, the prevalence of acute STEC infection is thought to be ∼2.8 million cases worldwide, with infection progressing to HUS in 3,890 cases and resulting in death in 230 cases (Majowicz et al., 2014).

**FIGURE 2:**
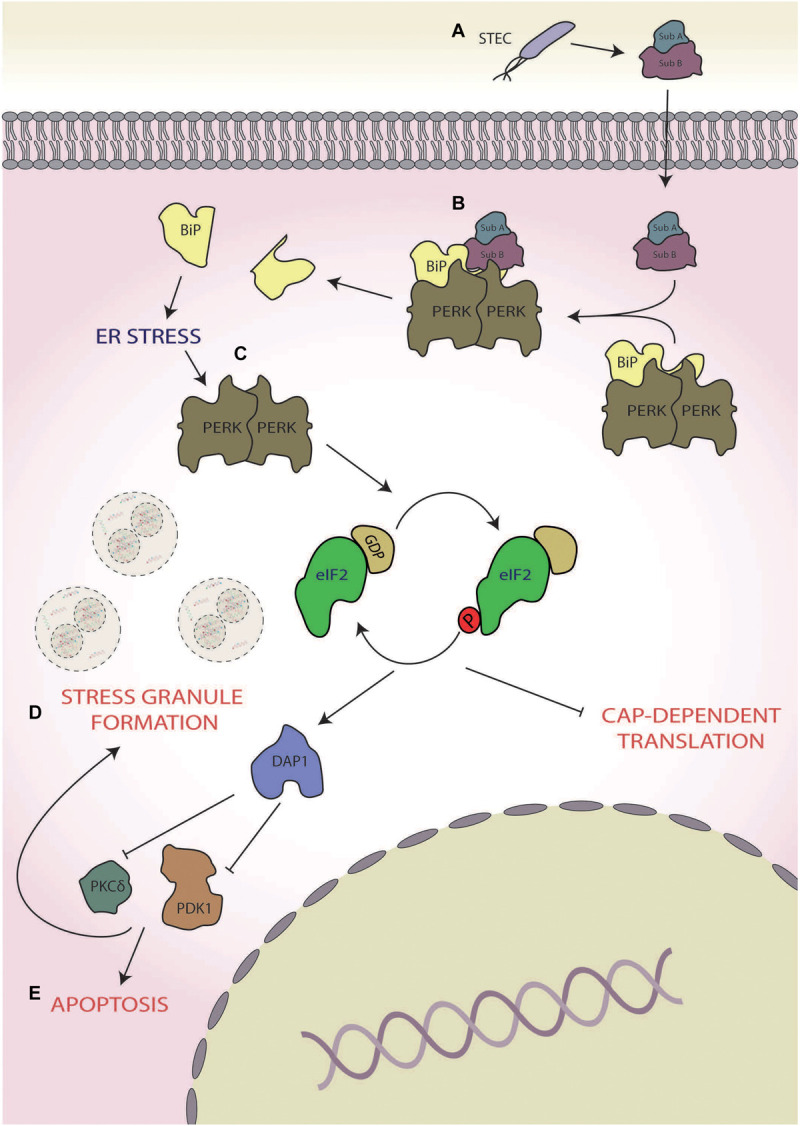
Shiga toxin-producing *Escherichia coli* (STEC). **(A)** During infection, STEC secretes subtilase cytotoxin (subAB), **(B)** which cleaves protein kinase R-like endoplasmic reticulum (ER) kinases (PERKs) chaperone binding immunoglobulin protein (BiP), and **(C)** leading to the activation of PERK and subsequent phosphorylation of eukaryotic initiation factor 2 alpha (eIF2α). **(D)** This results in the formation of stress granules in a manner dependent on death-associated protein 1 (DAP1) activation of phosphoinositide-dependent kinase 1 (PDK1) and protein kinase C δ (PKCδ), **(E)** pushing the cell towards death.

Virulent strains of STEC have been shown to target the ISR to induce host cell death mediated via a secreted virulence factor termed subtilase cytotoxin (SubAB; Tsutsuki et al., 2016; Figure 2A). SubAB is a secreted toxin consisting of two subunits; the B subunit binds the host extracellular toxin receptor and facilitates toxin internalisation, whereas the A subunit is a serine protease, which, in conjunction with the B subunit, facilitates the intracellular virulent effects of the pathogen (Morinaga et al., 2007). The main target of SubAB is the cleavage of the PERK chaperone binding immunoglobulin protein (BiP; Figure 2B), resulting in the dimerisation and activation of PERK, inducing eIF2α phosphorylation (Tsutsuki et al., 2016; Figure 2C). ISR activation triggered in this manner causes stress granule formation, which is dependent upon death-associated protein 1 activation (Tsutsuki et al., 2016). Inhibition of protein kinase C δ (PKCδ) and phosphoinositide-dependent kinase 1 (PDK1) are implicated in the formation of these stress granules, as chemical inhibition of both also heightens stress granule formation in response to SubAB (Tsutsuki et al., 2016). Furthermore, death-associated protein 1 knockdown increased basal levels of phospho-PDK1(S196), thereby inhibiting stress granule formation, further implicating PKD1 inhibition in formation of stress granules in response to SubAB (Tsutsuki et al., 2016; Figure 2D). Interestingly, in rat intestinal epithelioid cells, PDK1 has been shown to inhibit cell death in response to H_2_O_2_ (Song et al., 2009). Therefore, the inhibition of PDK1 coupled with prolonged PERK activation (Lin et al., 2009), which is known to promote apoptosis, may push the host cell towards death (Figure 2E; Lin et al., 2009; Tsutsuki et al., 2016). In contrast, in human lung cancer cells, PKCδ activation induces cell death via the CHOP-ATF3 arm of the ISR (Xu et al., 2012). Hence, the exact role PKCδ inhibition by SubAB in cell death requires further attention. Although driving the host cell towards death may seem counterproductive for bacterial survival, it is thought that Shiga-toxic *E. coli* displays altruism (Loś et al., 2012); in this context, ISR-mediated destruction of host cell and internalised bacteria could provide nutrients to the wider STEC community.

### Shigella flexneri

*Shigella* is a genus of gram-negative, facultative anaerobic bacteria that primarily infect the gastrointestinal tract, causing acute shigellosis (Fernandez and Sansonetti, 2003). Whilst closely related to *E. coli*, *Shigella* possesses unique methods of pathogenicity (Ud-Din and Wahid, 2014). Diarrhoea is an early symptom of infection as the bacteria moves through the small intestine, but the primary target of *Shigella* is the invasion of colonic epithelial cells from the basolateral surface (Phalipon and Sansonetti, 2007). Once internalised, the bacteria replicate and spread from cell to cell. The infection also causes inflammatory colitis via secreted toxins (Eashida et al., 2015). The mechanism of *Shigella* invasion has been reviewed elsewhere (Carayol and Van Nhieu, 2013; Liu et al., 2019).

**FIGURE 3:**
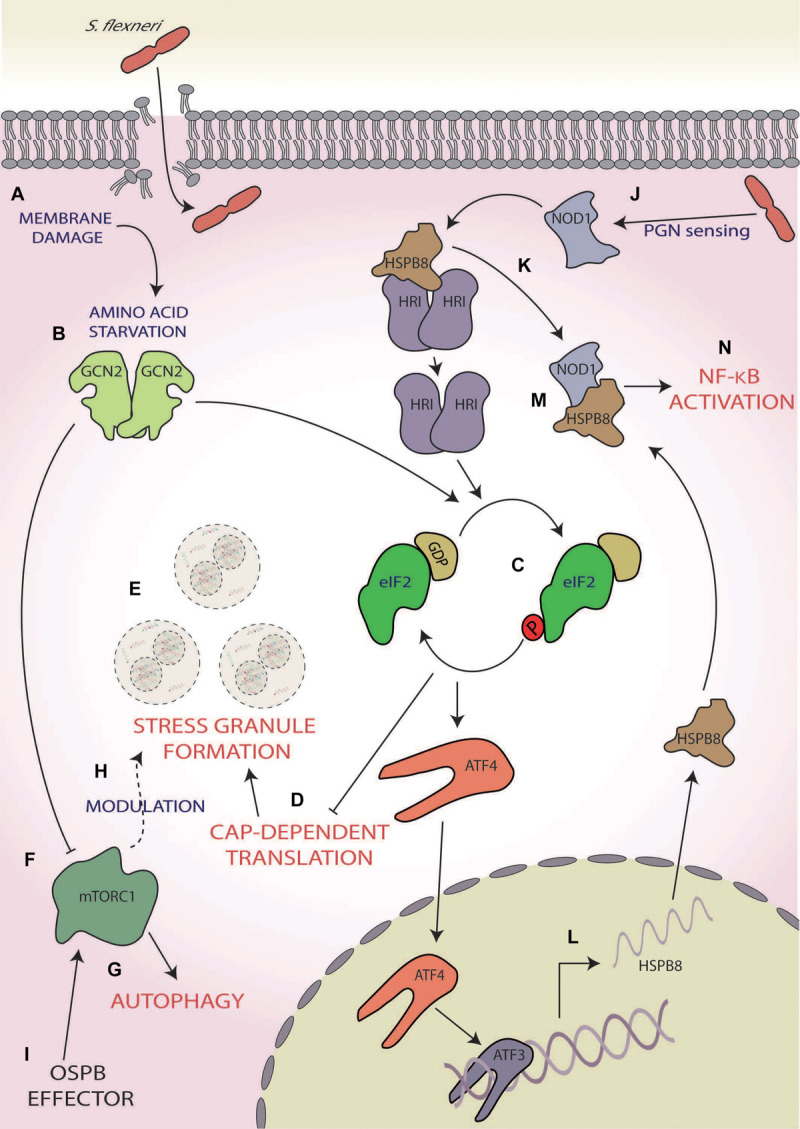
*Shigella flexneri.* **(A)** Membrane damage caused during *S. flexneri* internalisation induces amino acid (AA) starvation, **(B)** activating general control non-depressible 2 (GCN2), and **(C)** subsequently phosphorylation of eIF2α. **(D)** This results in the inhibition of cap-dependent translation initiation **(E)** and consequently the formation of stress granules. **(F)** GCN2 also inhibits mammalian target of rapamycin complex 1 [mTORC1; **(G)**], inducing autophagy and **(H)** modulating the frequency and composition of stress granules during exogenous stress induction. **(I)** mTORC1 activity is reactivated during later-stage infection via *S. flexneri’s* OspB effector. **(J)** Concurrently, *S. flexneri*’s peptidoglycan is detected by nucleotide-binding oligomerisation domain-containing protein 1 (NOD1), **(K)** which induces dissociation of the chaperone heat shock protein beta-r 8 (HSPB8) from HRI, causing its activation and subsequent eIF2α phosphorylation. **(L)** This results in the activation of activating transcription factor 4 (ATF4), which along with ATF3, upregulated the expression and translation of HSPB8. **(M)** This nascent HSPB8 associates with NOD1 **(N)** leading to the activation of pro-inflammatory responses by nuclear factor kappa-light chain-enhancer of activated B cell (NF-κB) activation.

Infection with Group B serogroup *S. flexneri* has been shown to robustly induce the ISR, resulting in the activation of two eIF2α kinases, GCN2, and HRI (Tattoli et al., 2012; Abdel-Nour et al., 2019; Figure 3). During the initial stage of infection, *S. flexneri* induces AA starvation through membrane damage, which results in the activation of GCN2 (Tattoli et al., 2012; Figure 3A). In its active form, GCN2 inhibits mTORC1 (Figure 3F), as demonstrated via its dispersal from LAMP2, and increases the transcription of the AA stress-related gene asparagine synthetase, a response that increases for up to 4 h post-infection (Tattoli et al., 2012; Abdel-Nour et al., 2019). However, *S. flexneri* is able to activate mTORC1 via direct delivery of its OspB effector into host’s cellular cytoplasm using the *S. flexneri* Type III secretion system (T3SS), which interacts with the IQ motif of the GTPase-activating protein 1 (Lu et al., 2015), an upstream regulator of mTORC1 (Tekletsadik et al., 2012; Figure 3I), ultimately resulting in increased host cell proliferation around the infection foci during the later stages of early infection. This increased cellular proliferation reduces *S. flexneri* spread but is thought to provide a preferential intracellular niche acting as a protective measure (Lu et al., 2015).

In addition, like STEC infection, *Shigella* infections also result in the aggregation of stalled messenger ribonucleoproteins (mRNPs) into stress granules (Tattoli et al., 2012; Vonaesch et al., 2016; Abdel-Nour et al., 2019; Figure 3E). The activation of the ISR leads to the upregulation of ATF3, ATF4, and GADD34 (Abdel-Nour et al., 2019) and consequently the robust upregulation of the transcription and expression of ISR and inflammatory-related genes (Tattoli et al., 2012; Abdel-Nour et al., 2019). Interestingly, in the presence of ISR-inducing exogenous stresses such as mitochondrial, oxidative and heat shock stress, an increase in frequency and a decrease in an area of stress granules formed are observed in *S. flexneri*-infected cells at 2–3.5 h post-infection (Vonaesch et al., 2016). The composition of the stress granules is also altered with the selective exclusion of eIF3B, eIF4G, and eIF4B via a mechanism downstream of eIF2α (Vonaesch et al., 2016). Since the phenotype observed is similar to the hindered assembly of stress granules seen following chemical disruption of the tubulin network with nocodazole (Fujimura et al., 2009; Kolobova et al., 2009; Vonaesch et al., 2016) and the movement of eIF3B and eIF4B is controlled by microtubule assembly (Shanina et al., 2001; Harris et al., 2006; Figure 3E), this differential stress granule composition may be dependent upon microtubule dysregulation. Interestingly, stresses such as selenite and hydrogen peroxide, which bypass eIF2α phosphorylation instead inhibiting mTORC1 function, also result in the formation of atypical stress granules lacking components such as eIF3 (Emara et al., 2012; Fujimura et al., 2012), thereby also implicating the *S. flexneri* infection-induced inhibition of mTORC1 in the differential stress granule formation (Tattoli et al., 2012; Vonaesch et al., 2016). As the formation of stress granules during *S. flexneri* infection has only been investigated up to 5 h (Abdel-Nour et al., 2019), whether the formation of stress granules is altered similarly during later-stage infection when mTORC1 is reactivated remains unknown. If this does not occur later when mTORC1 is reactivated, this would aid the hypothesis that modulation is at least partially dependent on mTORC1 inhibition. Furthermore, whether this modulation to stress granule formation during infection may provide any evolutionary benefit to *S. flexneri* or is simply a downstream effect of ISR modulation remains unknown.

In addition to ISR activation via membrane damage, intracellular sensing of bacterial peptidoglycan by the host PRR, nucleotide-binding oligomerisation domain-containing protein 1 (NOD1), also induces ISR activation and expression of the pro-inflammatory cytokine nuclear factor kappa-light chain-enhancer of activated B cells (NF-κB) in an HRI-dependent manner (Abdel-Nour et al., 2019; Figure 3J). NOD1 activation results in dissociation of the HRI chaperone, heat shock protein beta-r 8 (HSPB8), from HRI, consequently activating HRI (Abdel-Nour et al., 2019; Figure 3K). Activated HRI induces robust eIF2α phosphorylation (Figure 3C), resulting in heightened HSPB8 transcription in a manner dependent on ATF4 and ATF3 signalling (Abdel-Nour et al., 2019; Figure 3L). This nascent HSPB8 can interact with the previously dissociated HSPB8 and NOD1 to form a signalosome (Figure 3M) and during *S. flexneri* infection causes the upregulation of host immune inflammatory responses and macrophage activation through the NF-κB pathway (Abdel-Nour et al., 2019; Figure 3N). As this pathway is also triggered by misfolded proteins within the cytosol and is comparable with the UPR in the ER, it was coined the cytosolic UPR (cUPR; Abdel-Nour et al., 2019).

Shigella *flexneri* infection results in the induction of the ISR, which can be viewed as a protective response activating pro-inflammatory responses via the cUPR (Abdel-Nour et al., 2019) and autophagy via mTORC1 inhibition (Tattoli et al., 2012), during early infection. However, Abdel-Nour et al. (2019) found that the eIF2α S51A mutant, which cannot be phosphorylated, results in a significantly increased frequency of intracellular *S. flexneri* compared with cells with phosphorylated eIF2α (Abdel-Nour et al., 2019). Taken together, these data indicate that intracellular *S. flexneri* replication is heightened during ISR activation, and it is plausible that the phosphorylation state of eIF2α may at least partially control bacterial spread and viability.

However, during later-stage infection, *S. flexneri*-mediated reactivation of mTORC1 not only increases host cell viability but also decreases bacterial spread around the infection foci (Lu et al., 2015). Whilst the impact of mTORC1 reactivation on bacterial infection has not been investigated, focussing on the ISR may provide further insights into this mechanism. Furthermore, infection results in GADD34 expression; however, whether this occurs during later-stage infection, when mTORC1 is reactivated, is as of yet unknown (Abdel-Nour et al., 2019). As GADD34 induces the dephosphorylation of eIF2α via the activation of PP1 (Connor et al., 2001; Novoa et al., 2001), the potential of sustained GADD34 expression during later stage of *S. flexneri* infection (Abdel-Nour et al., 2019) may lead to termination of the ISR, potentially aiding the cell in returning to homeostatic conditions.

There is also evidence that inhibition of mTORC1 leads to eIF2α phosphorylation in cancer cell lines (Harvey et al., 2019). If this also occurs during *S. flexneri* infection, the reactivation of mTORC1 may further push the cellular equilibrium of eIF2α towards the non-phosphorylated form. Therefore, if dephosphorylation of mTORC1 was coupled with the GADD34 expression, this could act as a two-part shift to favour a state with minimal eIF2α phosphorylation. Though counter-intuitive, whilst defective eIF2α signalling favours *S. flexneri* invasion (Abdel-Nour et al., 2019), it may lead to increased host viability, which has been suggested to benefit *S. flexneri*, the latter remaining in infected cells for much of its life cycle (Killackey et al., 2016). Thus, whether mTORC1 reactivation or persistent GADD34 expression leads to eIF2α dephosphorylation during infection, and the consequential effects upon host cell viability and *S. flexneri* persistence and replication, is an area requiring further attention. Given that increased host cellular replication during OspB-mediated mTORC1 reactivation is thought to provide a preferential niche for *S. flexneri* survival (Lu et al., 2015), it is entirely plausible that this potential ISR termination may feed into this, helping to create an even further immune-privileged environment for *S. flexneri*. Furthermore, the effect of OspB only occurs later during the infection (Lu et al., 2015), whereas the initial phenotype of GCN2 activation and mTOR inhibition require internalisation and the resulting membrane damage (Tattoli et al., 2012; Abdel-Nour et al., 2019), which is intriguing as internalisation of OspB only requires extracellular contact between the bacteria and host cell (Hueck, 1998). Therefore, elucidation of the interaction between *S. flexneri* and these pathways may provide valuable insights into the pathogenic mechanisms of *S. flexneri* in chronic infections.

### Salmonella enterica

*Salmonella enterica* serovar Typhimurium is an enteric pathogen primarily associated with food-borne gastrointestinal disease (Fabrega and Vila, 2013) and is thought to affect 1.3 billion people annually, leading to approximately 3 million deaths globally (Pui et al., 2011). During infection, *Salmonella typhimurium* adheres to the host’s intestinal epithelium, resulting in extensive cytoskeletal rearrangements (Finlay et al., 1991). These modifications cause membrane ruffles, which eventually engulf the bacteria in large vesicles known as *Salmonella*-containing vesicles, creating an intracellular compartment in which *Salmonella* can survive and replicate (Steele-Mortimer, 2008). *Salmonella* infection is detected by host TLRs and NOD proteins, which initiates the NF-κB signalling cascade and results in cytokine and chemokine production, leading to an inflammatory state (Souvannavong et al., 2007; Spiller et al., 2008; Winter et al., 2009; Keestra et al., 2011).

**FIGURE 4:**
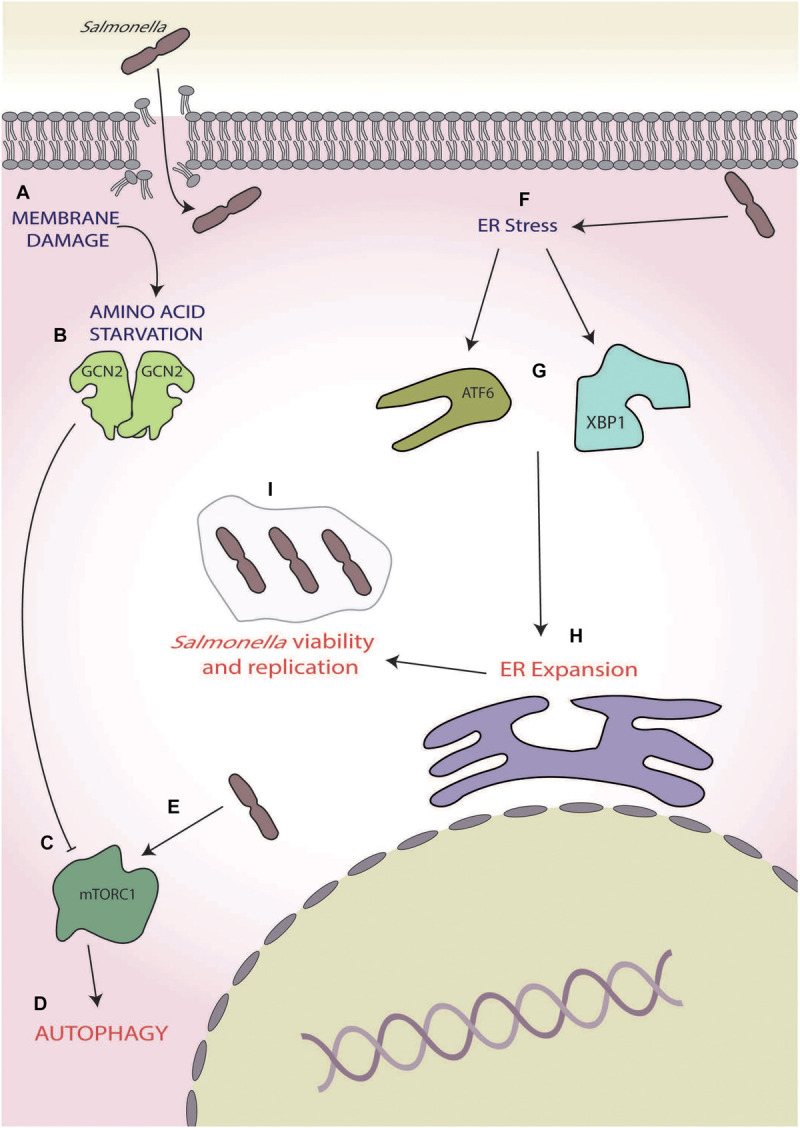
*Salmonella*. **(A)** Membrane damage caused during *Salmonella* internalisation induces amino acid (AA) starvation, **(B)** leading to general control non-depressible 2 (GCN2) activation, and **(C)** inhibition of mammalian target of rapamycin complex 1 (mTORC1), and **(D)** ultimately inducing autophagy. **(E)** During later-stage infection, *Salmonella* reactivates mTORC1, thereby inhibiting autophagy. **(F)** *Salmonella* also induces endoplasmic reticulum (ER) stress, **(G)** activating the unfolded protein response (UPRs) ATF6 and X-box binding protein 1 XBP1 arms, **(H)** leading to expansion of the ER, and **(I)** which increases intracellular *Salmonella* viability and replication.

As with *S. flexneri*, membrane damage induced by *Salmonella* invasion causes intracellular AA starvation with the activation of ISR through the eIF2α kinases GCN2 (Tattoli et al., 2012; Figures 4A,B). This AA starvation-induced activation of GCN2 also initially inhibits the activity of mTORC1, via the dispersal from LAMP2 and results in the activation of autophagy (Tattoli et al., 2012; Figures 4C,D). Interestingly, within 4 h of infection, the raptor/rag/regulator pathways can reactivate mTORC1, effectively saving *Salmonella* from autophagy; however, the mechanism by which this occurs has yet to be fully determined (Tattoli et al., 2012; Figure 4E).

In later stages of infection, e.g., 12–24 h post-infection, *Salmonella* has also been shown to induce ER stress (Figure 4F), robustly activating the UPR and leading to the activation of X-box binding protein 1 and transcription factor 6 (Antoniou et al., 2018; Figure 4G), which is known to increase lipid biogenesis and increase ER expansion (Sriburi et al., 2004, 2007; Figure 4H). During *Salmonella*-induced ER stress, human leukocyte antigen (HLA)-B27 becomes misfolded, causing SCV to move away from host golgi apparatus (Antoniou et al., 2018). This, coupled with ER expansion, is thought to allow for increased space for the SCV and is supported by observations that ER stress induction by thapsigargin and misfolded HLA-B27 increase intracellular *Salmonella* viability and replication (Antoniou et al., 2018; Figure 4I). Thus, *Salmonella* effectively utilises the ISR and UPR in two opposing ways, firstly by reversing the autophagic responses brought on by the ISR during early-stage infection via mTORC1 reactivation and then inducing ER stress to allow for preferential replication conditions in later stages of infection.

### Pseudomonas aeruginosa

*Pseudomonas aeruginosa*, a gram-negative, rod-shaped, mono-flagellated bacterium, is one of the most frequent causative agents for hospital-acquired infections resulting in loss of life (Buhl et al., 2015), with immunocompromised patient’s survival rates being disproportionately lowered (Migiyama et al., 2013). Chronic lung infections caused by *P. aeruginosa* are a common cause of death in patients with cystic fibrosis and chronic obstructive pulmonary disease, with those affected often experiencing recurrent infections (Murphy et al., 2008; Yum et al., 2014).

**FIGURE 5:**
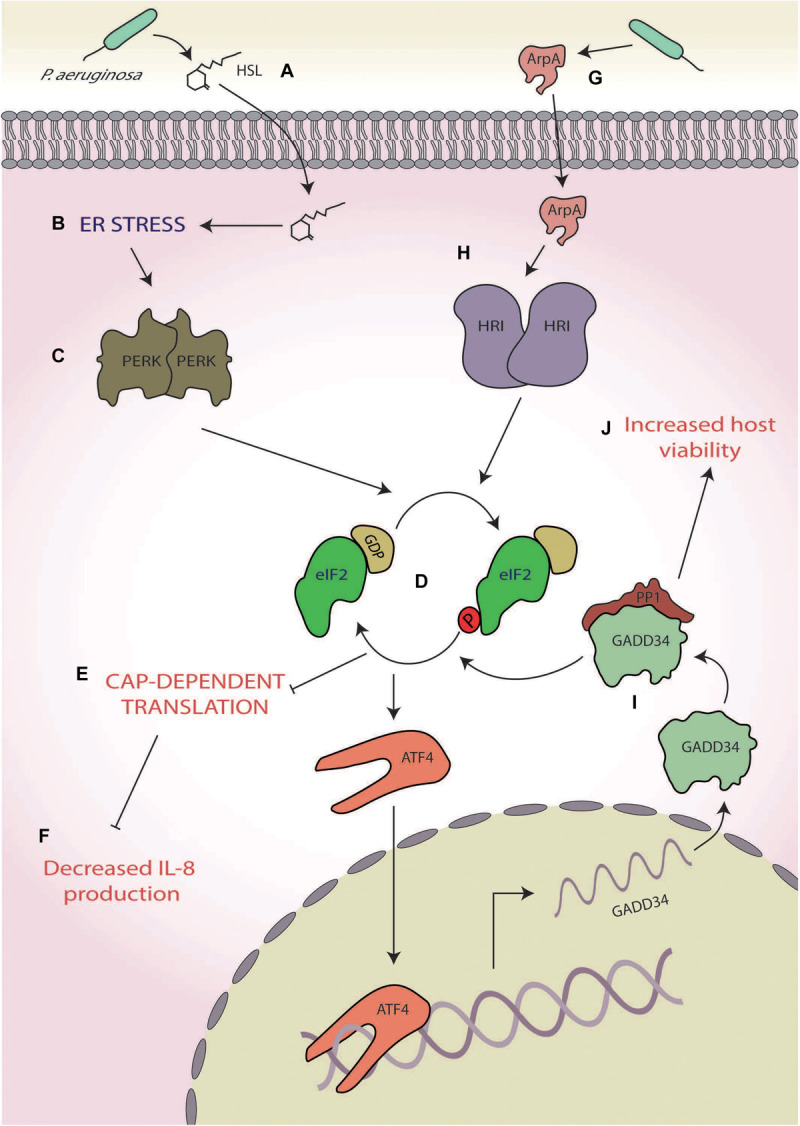
*Pseudomonas aeruginosa*. **(A)** The quorum-sensing molecule *N*-(3-oxododecanoyl)-homoserine lactone (HSL) secreted by *P. aeruginosa* **(B)** induces endoplasmic reticulum (ER) stress, **(C)** activating protein kinase R-like ER kinases (PERKs), and **(D)** which leads to eukaryotic initiation factor 2 alpha (eIF2α) phosphorylation. **(E)** The consequential translational stalling **(F)** results in decreased expression of the pro-inflammatory cytokine interleukin 8 (IL-8) production. **(G)** A protease, alkaline protease A (ArpA), secreted by *P. aeruginosa* **(H)** activates heme-regulated inhibitor (HRI), **(I)** leading to the specific upregulation of growth arrest and DNA damage-inducible protein (GADD34), and **(J)** which increases host cell viability.

During infection, *P. aeruginosa* secretes a wide variety of proteins including the extracellular adhesin CdrA (Borlee et al., 2010), the diffusible quorum-sensing molecule *N*-(3-oxododecanoyl)-homoserine lactone (HSL; Smith et al., 2002), and virulence factors [e.g., alkaline protease A (ArpA; Vasil and Ochsner, 1999) and HasAp (Létoffé et al., 1996)], all of which are known to induce ER stress (Grabiner et al., 2014; van‘t Wout et al., 2015; Figure 5A). In mouse embryonic fibroblasts (MEFs), HSL, which has a key role in *P. aeruginosa* cell-to-cell communication within the structurally ordered biofilm (Smith et al., 2002), induces ER stress via the release of Ca^2+^ from ER stores (Figure 5B). This causes an imbalance in ER homeostasis and activates PERK (Figure 5C), which phosphorylates eIF2α and results in a global shutdown of protein synthesis (Grabiner et al., 2014; Figures 5D,E). Interestingly, this translational stalling reduces the expression and secretion of the pro-inflammatory chemokine keratinocyte chemoattractant (KC), the mouse equivalent of interleukin 8 (IL-8; Grabiner et al., 2014; Figure 5F). Thus, HSL may lead to the suppression of KC secretion through eliciting the host ISR, aiding *P. aeruginosa* to evade host inflammatory and antibacterial responses during the early stages of infection (Grabiner et al., 2014). This observation contrasts with the robust expression of IL-8 seen in *S. flexneri* infection (Abdel-Nour et al., 2019) and suggests a species-specific response (Abdel-Nour et al., 2019). However, further studies are required to probe this and ascertain whether this response is species specific or cell dependent due to the differential approaches used with one study using mouse-derived MEFs (Grabiner et al., 2014) and the other using the human cervical epithelial cell line HeLa (Abdel-Nour et al., 2019).

Another example of *P. aeruginosa*-mediated ISR manipulation is through the secretion of ArpA (Figure 5G), a protease involved in, amongst other pathways, hosts siderophore-mediated iron scavenging (Vasil and Ochsner, 1999; Kim et al., 2006; van‘t Wout et al., 2015). Whereas HSL induces ER stress through activation of the p53 MAPK pathway, ArpA specifically activates HRI (Figure 5H), which induces the expression of GADD34 (van‘t Wout et al., 2015; Figure 5I) and is protective against *P. aeruginosa* cytotoxicity, allowing for prolonged host cell survival (van‘t Wout et al., 2015; Figure 5J). The mechanism by which HRI activation and GADD34 expression increase host viability is currently unknown, but increased GADD34 expression could lead to increased PP1 activity and consequently dephosphorylation of eIF2α (Connor et al., 2001; Novoa et al., 2001). This deactivation of the ISR could prove to be a promising system for *P. aeruginosa* to push the cell towards survival, thereby increasing viability. However, given the recent findings of Abdel-Nour et al. (2019), it is entirely plausible that activation of HRI by ArpA may activate the cUPR, which has been shown to be protective against *S. flexneri* infection. Whether this increased viability is due to eIF2α dephosphorylation or cUPR activation, or a combination of both, remains to be elucidated and requires further attention.

*Pseudomonas aeruginosa* hereby displays a two-part manipulation of the host ISR, both dampening inflammatory responses and increasing host cell viability. These reduced inflammatory and immune responses may act as the critical tipping point, leading to the decreased survival of immunocompromised patients (who already have impaired immune responses), as it could result in unregulated and therefore heightened *P. aeruginosa* growth.

### Porphyromonas gingivalis

*Porphyromonas gingivalis* is a gram-negative, anaerobic bacterium and the “keystone pathogen” of the chronic oral inflammatory gum disease, periodontitis (Socransky et al., 1998). Infection triggers host immune responses resulting in inflammation of the gingival tissues, which in some cases progresses to periodontitis, resulting in alveolar bone resorption and ultimately tooth loss (Pihlstrom et al., 2005). *P. gingivalis* is known to modulate several host cell responses including the inhibition of antimicrobial responses whilst leaving pro-inflammatory signalling active, thereby providing nutrients from inflammatory spoils (Hajishengallis and Lambris, 2012). To achieve this, *P. gingivalis* employs a range of virulence factors including lipopolysaccharides (LPSs), fimbriae and lysine- and arginine-specific cysteine proteases, termed gingipains (Jia et al., 2019). Gingipains are cell surface-anchored proteins (Andrian et al., 2006), which can also be excreted in membrane-bound vesicles (Grenier et al., 1989) and therefore can account for up to 85% of proteolytic activity around the *P. gingivalis* infection site (De Diego et al., 2013).

**FIGURE 6:**
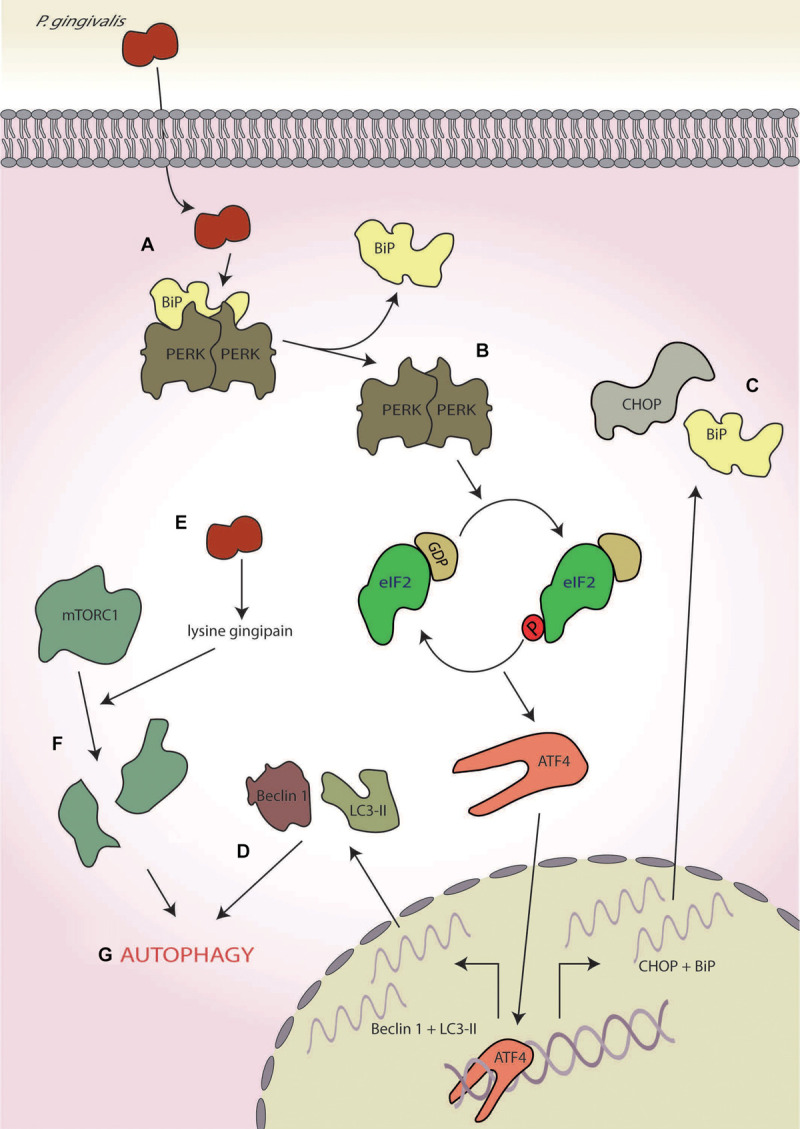
*Porphyromonas gingivalis*. **(A)** *P. gingivalis* infection induces endoplasmic reticulum (ER) stress, **(B)** leading to protein kinase R-like ER kinase (PERK) activation, and **(C)** the expression of C/EBP homologous protein (CHOP) and binding immunoglobulin protein (BiP). **(D)** Concurrently, it also induces to autophagy as determined by the markers Beclin-1 and 1A/1B-light chain 3 (LC3-II). **(E)** *P. gingivalis* secretes a lysine-specific cysteine protease, termed the lysine gingipain, **(F)** which degrades mammalian target of rapamycin complex 1 (mTORC1), and **(G)** leading to the induction of autophagy.

A recent study using human umbilical vein cells as host cells suggested that the virulence of *P. gingivalis* (strain 381) may involve the UPR and ISR (Hirasawa and Kurita-Ochiai, 2018; Figure 6). In this study, the authors showed that whilst infection ultimately resulted in apoptosis after 21-h infection, early-stage infection (∼8 h) resulted in ER stress characterised by increased expression of CHOP and BiP at both the transcriptional and translational levels coupled with increased caspase-12 activity (Hirasawa and Kurita-Ochiai, 2018; Figures 6A–C). In addition, enhanced autophagy, characterised by the increased expression of autophagy markers Beclin-1, microtubule-associated protein 1A/1B-light chain 3, and acidic vesicular organelles (Figure 6D), was also observed. This response was inhibited by pretreatment with an ER stress inhibitor salubrinal, an inhibitor of PP1, that results in blockage of eIF2α dephosphorylation (Boyce et al., 2005). Furthermore, siRNA knockdown of LC3 resulted in increased apoptosis, thereby implicating ER stress-induced autophagy as a protective response against *P. gingivalis*-induced apoptosis (Hirasawa and Kurita-Ochiai, 2018). These results are corroborated by studies in mice where administration of *P. gingivalis* induced ER stress with increased expression of both CHOP and BiP (Yamada et al., 2015).

Interestingly, the lysine-specific gingipain of *P. gingivalis* has been shown to degrade mTORC1 and modulate levels of mTORC1-associated proteins in oral epithelial cells after 4 h of infection (Stafford et al., 2013; Figures 6E,F). However, this mTOR degradation requires *P. gingivalis* internalisation, indicating that these effects are probably not mediated by the secretory fraction of gingipains produced by extracellular *P. gingivalis* (Stafford et al., 2013). Inactivation of mTOR is known to induce autophagy (Jung et al., 2010), fitting with the early stage autophagy seen by Hirasawa and Kurita-Ochiai (2018; Figure 6G). Furthermore, mTOR inhibition by rapamycin suppresses tunicamycin-induced ER stress, resulting in autophagy (Dong et al., 2015). Therefore, gingipain-mediated degradation of mTOR may help dampen ER stress induced by *P. gingivalis* infection, aiding host cell survival in the early stages of infection by delaying the onset of apoptosis.

## Discussion and Future Perspectives

Infection by pathogens triggers concerted whole organism immune responses by the host, which are often initiated at the cellular level. In fact, individual host cells can respond independently to adverse conditions via a variety of intracellular signalling systems, with the ISR being a key mediator of these responses and determining cellular fate (Costa-Mattioli and Walter, 2020). In recent years, it has become apparent that the ISR may have a wider role in host immune responses (Cláudio et al., 2013; Pulendran, 2015). Here, we discuss recent advances in understanding host–microbe interactions, which demonstrate that bacterial pathogens can interact with the host ISR during infection, directly manipulating cellular fate and immune signalling. This demonstrates that a comprehensive understanding of pathogenic interactions with the ISR is crucial for the elucidation of microbial disease progression.

Given the paradoxical role of the ISR, any change to a particular signal can have vastly different outcomes dependent upon the circumstance. For example, in neuronal cells, inhibition of PERK is protective during stress induction (Moreno et al., 2013); conversely in pancreatic cells, it induces type I interferon activation, proving to be fatal (Yu et al., 2015). These opposing outcomes of ISR dysregulation are also apparent during bacterial infection. Where PERK activation by STEC and *Porphorymonas gingivalis* infection ultimately leads to cell death (Tsutsuki et al., 2016; Hirasawa and Kurita-Ochiai, 2018), PERK activation by *P. aeruginosa* reduced the secretion of the pro-inflammatory cytokine KC, the mouse equivalent of IL-8, potentially aiding immune evasion (Grabiner et al., 2014). Conversely, *S. flexneri* infection and activation of both GCN2 and HRI showed the opposite of this phenotype, leading to robust upregulation of IL-8 (Abdel-Nour et al., 2019). Although it remains to be elucidated whether these changes are cell type or infection specific, these conflicting phenotypes demonstrate the range of outcomes that bacterial manipulation of the ISR can have on immune signalling and cellular fate; however, further work is required to evidence this.

Furthermore, Abdel-Nour et al. (2019) found that inhibition of eIF2 signalling via the knock-in eIF2α S51A mutant induced a significant decrease in intracellular *S. flexneri*, thereby implicating eIF2α signalling in the control of bacterial internalisation. These findings corroborate those of Shrestha et al. (2012), who reported that invasion of the Far East scarlet-like fever causing pathogen *Yersinia pseudotuberculosis* resulted in a 25-fold increase in MEFs containing the eIF2α S51A knock-in compared with wild type. The authors also identified functional eIF2α signalling as a prerequisite for cytokine expression and demonstrated that *Y. pseudotuberculosis* specifically dampens eIF2 phosphorylation during a range of cellular stresses through the action of a virulence factor YopJ, which is inserted directly into host cells via a T3SS. Ultimately, this resulted in decreased pro-inflammatory cytokine expression (Shrestha et al., 2012). These findings further demonstrate the potential for ISR manipulation as an immuno-evasive mechanism of bacterial pathogens.

Particularly striking is that both *S. flexneri* and *P. aeruginosa* infection result in the activation of HRI, with *S. flexneri* inducing HRI activation leading to initiation of immune signalling as demonstrated by NF-κB activation (Abdel-Nour et al., 2019) and *P. aeruginosa* inducing HRI activation leading to increased host cell viability (van‘t Wout et al., 2015). Interestingly, during monospecies bacterial infection, HRI activation is required for *Y. pseudotuberculosis* and *Listeria monocytogenes* to achieve their virulence associated intracellular activities, where a lack of HRI interferes with the pathogens T3SS virulence factors (Shrestha et al., 2013). In contrast, at an organismal level, HRI-deficient mice have been shown to be more susceptible to *L. monocytogenes* infection and less able to mount a system-level cytokine response (Bahnan et al., 2018). This suggests potentially different outcomes depending on whether infection is monospecies or polymicrobial in nature, and hence, the exact role of HRI needs further attention. To date, HRI-specific inhibitors have been identified (Rosen et al., 2009), and PERK inhibitors have already shown promise in combatting neurodegenerative diseases that impact upon the ISR (Moreno et al., 2013). Greater understanding of the exact role of HRI in bacterial infection is therefore an area that may allow for the targetting of HRI as a novel antimicrobial therapy using inhibitors or activators in an infection-specific manner. Findings that internalisation efficiency of *Chlamydia trachomatis* was independent of HRI activity and that it was increased by loss of PKR (Shrestha et al., 2013) support PKR as another potential therapeutic target.

During prolonged ER stress, LPS is known to trigger a TLR-dependent reprogramming of the ISR (Woo et al., 2012). LPS is detected by TLRs, which triggers a signalling cascade mediated by the action of its downstream adaptor TRIF and results in decreased serine phosphorylation of eIF2Bε, thereby increasing eIF2B GEF activity in a manner independent of eIF2α phosphorylation (Woo et al., 2012). This increased eIF2B GEF activity results in suppression of CHOP, increasing cell survival, and increased translation of the pro-inflammatory cytokine TNF-α (Woo et al., 2012). Both *P. aeruginosa* and *P. gingivalis* induce the production of host TNF-α dependent on their LPS (Raoust et al., 2009; Nativel et al., 2017). Therefore, given that both *P. aeruginosa* and *P. gingivalis* infections are long term and chronic and can induce ER stress and PERK activation, it is entirely plausible that the TNF-α expression may be at least partially dependent on the TLR/TRIF eIF2B pathway (Grabiner et al., 2014; van‘t Wout et al., 2015; Yamada et al., 2015; Hirasawa and Kurita-Ochiai, 2018). Indeed, *P. gingivalis* survival is known to hinge upon increased inflammatory signalling, whilst dampening host antimicrobial responses, all in a TLR-dependent manner (Hajishengallis and Lambris, 2012). Here, investigation into this potential reprogramming of the ISR during infection may yield crucial information into *P. gingivalis* virulence and may point to the potential of therapeutic targeting of eIF2B activity during chronic infection. Given that Woo et al. (2012) only investigated the TLR-dependent ISR reprogramming under ER stress, it is entirely possible that this signalling cascade may also occur during other stresses; therefore, all of the bacteria discussed above may induce this response. The limiting factors would be host cell survival time and cytotoxicity of infection, as this cascade occurs primarily during long-term stress (Woo et al., 2012).

This review has highlighted a diverse range of cellular outcomes during bacterial manipulation of the ISR. It should be noted that most of the studies to date have investigated the role of a single species upon a single cell type, whereas most bacterial infections are polymicrobial (Brogden et al., 2005). As with host immune responses, pathogenic bacteria are known to interact with other microbes; indeed, the virulence and disease severity of both *P. gingivalis* and *Salmonella* infection are thought to be reliant upon their ability to manipulate the wider bacterial community (reviewed in Hajishengallis et al., 2012). *P. aeruginosa* is also known to secrete products, which have a community wide effect in cystic fibrosis patients, ultimately shaping microbial community dynamics within the lung (Reviewed in O’Brien and Fothergill, 2017). Whilst, pyocyanin, a quorum-sensing molecule secreted by *P. aeruginosa* in response to gram-negative cell wall fragments, is thought to reduce microbial community diversity to select for a more pathogenic community (Norman et al., 2004; Korgaonkar and Whiteley, 2011; Korgaonkar et al., 2013), pyocyanin also functions to generate ROS (Xu et al., 2013), a known inducer of the ISR. Given that secretion of pyocyanin is governed by inter-bacterial communication, which is inherently non-linear (Dietrich et al., 2006), alterations in pyocyanin concentrations could induce differential ROS production over time. This could plausibly result in oscillation of host ISR activation, adding another layer of complexity to the ISR dampening interaction seen during monospecies *P. aeruginosa* infection. Furthermore, *Bifidobacterium* spp. protects mice from STEC toxicity via the production of acetate, which inhibits the subAB toxin produced by STEC (Fukuda et al., 2011, 2012). This lowering of toxicity may well be due dampening of STEC-mediated ISR activation in host tissues around the infection sites, especially as subAB self-internalises (Morinaga et al., 2007), which is likely be in contact with the extracellular acetate before the internalisation event. Therefore, given the role of the wider bacterial community upon virulence, studying the interactions between polymicrobial communities and the host ISR may lead to advances in the understanding of host–pathogen interactions, reflect physiological conditions and act as a platform for possible therapies.

## Author Contributions

AK, SC, NC, and PS conceived and planned the manuscript. AK wrote the manuscript and designed the figures. AK, SC, NC, and PS edited and revised the manuscript. All authors approved and revised the final version of the manuscript.

## Conflict of Interest

The authors declare that the research was conducted in the absence of any commercial or financial relationships that could be construed as a potential conflict of interest.
